# Arid habitats intensify sexual conflict in invasive cane toads (*Rhinella marina*)

**DOI:** 10.1098/rsos.251341

**Published:** 2025-11-12

**Authors:** Richard Shine, Georgia Ward-Fear, Chris James Jolly, Terri Shine, Antoine Païta, Alexander Funk

**Affiliations:** ^1^ School of Natural Sciences, Macquarie University, Sydney, New South Wales, Australia; ^2^ Charles Darwin University, Darwin, Northern Territory, Australia; ^3^ ENS de Lyon Département de Biologie, Lyon, Auvergne-Rhône-Alpes, France

**Keywords:** *Bufo marinus*, Bufonidae, costs of reproduction, invasive species, mating behaviour

## Abstract

Amplexus by male cane toads (*Rhinella marina*) impairs a female’s mobility and may impose a risk of drowning. Near the arid-zone edge of the toads’ Australian invasion, artificial ponds provide the only permanent open water. Cane toads must access water to hydrate every few nights, creating a potential for sexual conflict. Our field-based experiments show that a female toad that approaches one of these steep-sided dams encounters numerous reproductively active males, most of which are facing the shore. When amplexed by these males, she may find herself in deep water even close to the shore and is vulnerable to drowning. In trials with tethered females, toads amplexed in deep water could not hold their heads above the water’s surface. Demographic effects of this sexual conflict are evident from population surveys: toad populations around dams are strongly male-biased whereas females are concentrated at mesic refuges around buildings that provide less dangerous conditions. Even around the same dam, female toads are often found on land whereas most males are found in the water. If sexual conflict around scarce waterbodies is lethal for female toads, we might reduce recruitment by allowing dense populations of male toads to persist.

## 1. Introduction

Even within a single population, adult males and adult females often differ substantially not only in morphology and physiology but also in ecological traits such as diets, habitat use and seasonal and diel patterns of activity [1]. Those ecological divergences may arise through both direct and indirect processes. One common indirect process involves ecological divergence that arises as a by-product of sexual dimorphism in body sizes and shapes. For example, reproduction-related advantages to larger body size in males (via sexual selection for the ability to overpower rivals) or females (via enhanced fecundity) may favour the evolution of different adult body sizes in the two sexes—thereby rendering one sex more capable of exploiting specific food resources (e.g. large prey, in a gape-limited predator) or microhabitats (e.g. narrow burrows or thin tree branches) than the other [2]. Alternatively, ecological divergence between the sexes may confer direct advantages: for example, males aggregate at sites that provide access to mating opportunities whereas females aggregate at sites that provide nesting opportunities [1]; or females obtain nutrients for offspring production by foraging in particular places [3,4]. Lastly, ecological divergence between the sexes may be driven by sexual conflict, whereby one sex (often, females) benefits from avoiding the other sex (often, males) because of fitness decrements due to harassment (e.g. coercive attempts to mate [5,6]) and/or to competition for resources due to depressed food availability in sites with high densities of the other sex [7].

Teasing apart the mechanisms that result in sex-based ecological divergence is difficult, because of those multiple drivers, but can be valuable for attempts to manage wildlife populations. For example, sites in which reproductive females aggregate (rookeries, communal-nesting areas) are disproportionately important for population-level recruitment. Such sites should thus be protected, if our aim is to conserve an endangered species; or heavily culled, if we wish to eradicate an invasive species [8,9]. More generally, an understanding of sex-specific patterns of habitat use, and of the mechanisms responsible for those patterns, can inform management as well as clarify fundamental ecological questions about intraspecific variation in ecological traits.

**Figure 1.**
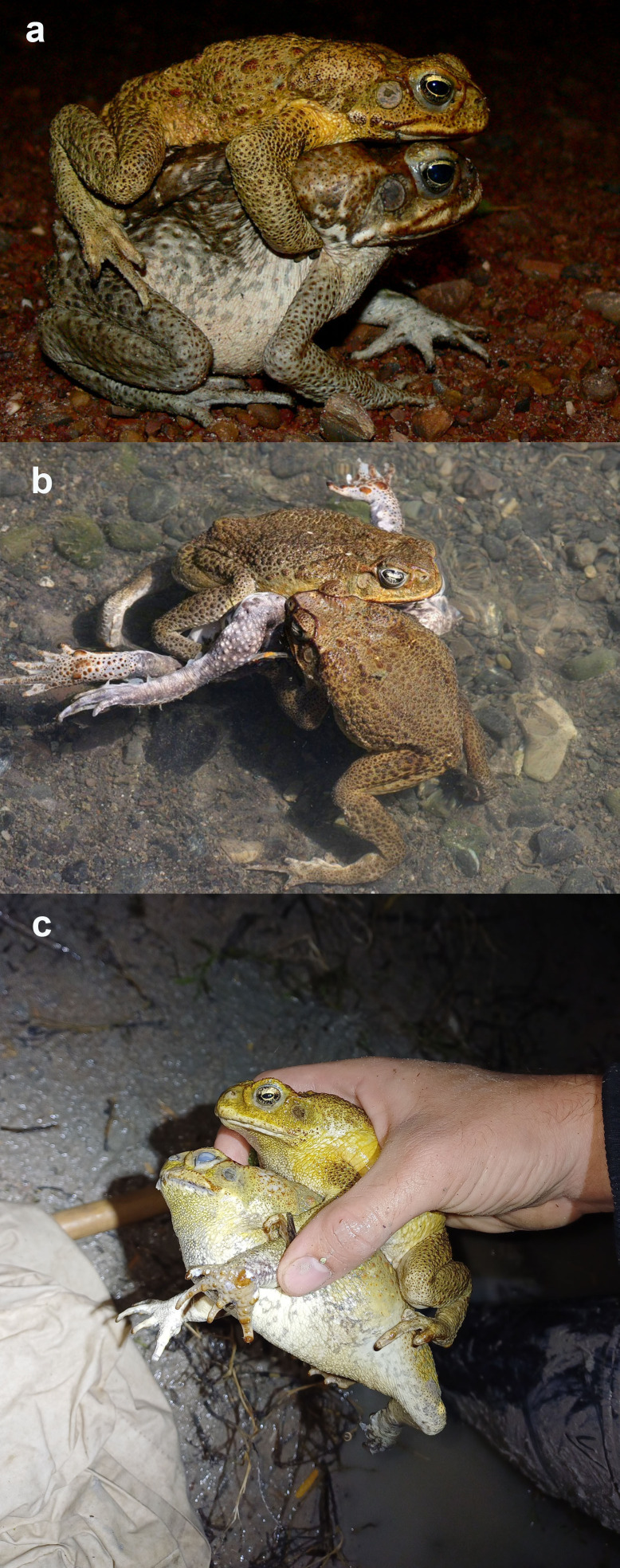
(a) Amplexus in cane toads (photo by C. Kelehear); (b) female cane toad drowned by multiply amplexing males (photo by W. Hull, iNaturalist); (c) dead female toad being amplexed by live male (photo by A.P.).

Invasive species offer powerful opportunities to explore this topic because the spatial and temporal distribution of resources often differ between a species’ native range and its invaded range. Thus, pre-existing patterns of sex-specific habitat use may be exaggerated (or reduced) by the shift to novel ecological conditions, and very high population densities in the invaded range (likely due to escape from coevolved pathogens, predators and competitors [10]) may exacerbate the frequency and intensity of interactions between the sexes. This situation has arisen during the invasion of tropical Australia by cane toads (*Rhinella marina*; *Bufo marinus* in earlier literature). In many pond-breeding anuran amphibians, reproductively active males tend to remain close to a waterbody whereas females are found through the broader landscape [11]. That sex-based divergence in habitat use is seen in mesic areas within the cane toad’s invaded range in Australia [12,13] and probably within the native range also (based on data for the congeneric *R. horribilis* in Central America [14]) and likely reflects reproduction: cane toads lay their eggs in still water, and the tadpoles are aquatic. Because spawning is restricted to open-water sites, males maximize their rates of encounter with reproductive females by remaining close to those waterbodies [12,13]. The resultant high densities of toads around waterbody margins likely depress the availability of insect prey [15], such that females can forage more productively in areas away from the pond [12,16]. However, sexual harassment may also favour female avoidance of male-dominated sites. Amplexus (figure 1a) compromises a female toad’s mobility and her ability to feed [17] and in the extreme may result in females being drowned [18,19] (see below).

The potential costs of harassment for a female cane toad likely vary among habitat types. Unlike native Australian anurans from arid-zone habitats, cane toads need to rehydrate frequently (once every few nights [20,21]). In the species’ native range and in much of the Australian invaded range, rehydration opportunities on damp soil and widely distributed puddles of water are plentiful—but the toads’ invasion has also taken them into hot, dry habitats where the only surface water is concentrated in artificial dams for most of the year [22,23]. Because those dams support large numbers of reproductively active male toads, any female approaching the dam to hydrate may face increased risk of harassment, and even drowning. We might thus expect limited water availability in this landscape to exacerbate sexual conflict in invasive cane toads. To explore this possibility, we gathered data on:

(1) *Attributes of ponds that might affect a female’s vulnerability*. We predict that visiting a pond is risky for a female if (i) the banks are steep (rendering it difficult for a female to move away from water if amplexed; and more likely for her to fall into the water if amplexed by multiple males), and (ii) the water is deep even close to the shoreline, with little aquatic vegetation to which a female could cling.
(2) *Effects of amplexus on females*. We predict that deeper water translates into a higher risk of drowning for a female that is being amplexed by a male.
(3) *Operational sex ratios (OSRs)*. We predict that visiting a pond is risky for a female if males greatly outnumber females.
(4) *The locations of adult male toads*. We predict that visiting a pond is risky for a female if males line the shoreline and can intercept approaching females.
(5) *Distribution of sexes across the landscape*. We predict that females avoid ponds in favour of other habitat types (drainage lines, anthropogenically watered areas) that provide damp substrates where they can hydrate without the risk of drowning.
(6) *Distribution of sexes around a pond*. We predict that male toads will hydrate in the open water of the pond whereas females will more often use damp terrestrial sites for this function.

## 2. Methods

### 2.1. Study species and sexual conflict

Cane toads are large and highly toxic bufonid anurans native to South America that have been translocated in futile attempts at pest-insect biocontrol [24–26]. Brought to northeastern Australia in 1935, toads have now spread widely across the tropics and subtropics, fatally poisoning native predators [25]. In Australia, the toad’s westward invasion has taken them into areas that are much hotter and seasonally drier than the relatively mesic South American habitats of their native range [27]. Unlike many native Australian anurans, cane toads remain active year-round if ambient temperatures are high, and individuals must rehydrate every few nights in water or on moist soil [21,28].

Female cane toads grow larger than males, but the sexes overlap in adult body sizes. At our study sites, mean snout–urostyle lengths (SULs) were 105.3 mm (SE = 0.31, range 81−126 mm SUL) for adult males and 109.0 mm (SE = 0.70, range 81−139.0 mm SUL) for adult females. For mass, males averaged 146.0 g (SE = 1.19, range 32−309 g) and females averaged 167.5 g (SE = 2.83, range 35−620 g). Reproductively active male cane toads seize females (or other males, and sometimes frogs and other objects [29–31]) with inguinal amplexus (figure 1a); occasionally, a female is seized by more than one male at a time (figure 1b).

The most significant cost of amplexus for a female anuran may be the risk of drowning, especially if she is amplexed simultaneously by multiple males in deep water. There are many reports of female toads (and frogs) being drowned in such circumstances, beginning with Charles Darwin’s note (in *Sexual selection and the descent of man*, 1871, p. 1036 [32]) that ‘*Dr. Gunther informs me that he has several times found an unfortunate female toad dead and smothered from having been so closely embraced by three or four males’*. We are not aware of any quantitative data on the frequencies of female mortality due to amplexus, but many authors have reported the phenomenon [18,33] (and see figure 1c for an example from our own study). Gray & MacKenzie [34] saw an amplectant pair of cane toads roll down a steep rockface. Sathyanarayana & Ganesh [35] reported finding several groups of up to 20 dead female Asian toads (*Duttaphrynus melanostictus*), apparently mass drowning events during amplexus, and reviewed published accounts of male toads found amplexing drowned females.

### 2.2. Study area

In the Northern Territory of tropical Australia, invasive cane toads occupy a broad range of habitats from monsoonal savannah in the north to semi-arid rangelands in the south. Toads are distributed widely across mesic habitats, but their need for frequent access to water for rehydration restricts them to isolated pockets of moist conditions in semi-arid areas [21,23,28]. We worked in an area near the towns of Daly Waters, Dunmarra and Elliot along the Stuart Highway, 590−735 km south of the city of Darwin. Maximum daily ambient temperatures are high year-round (at Daly Waters, mean maxima > 30°C in 10 months per year) but with low minimum temperatures at night during the mid-year dry season (May to August, monthly mean minima 11.8−15.8°C) (http://www.bom.gov.au/climate/averages/tables/cw_014618.shtml). Annual rainfall is relatively low (mean at Daly Waters: 681 mm) and is concentrated in the warmest months (median of zero rainfall from May to September) (http://www.bom.gov.au/climate/averages/tables/cw_014618.shtml).

**Figure 2.**
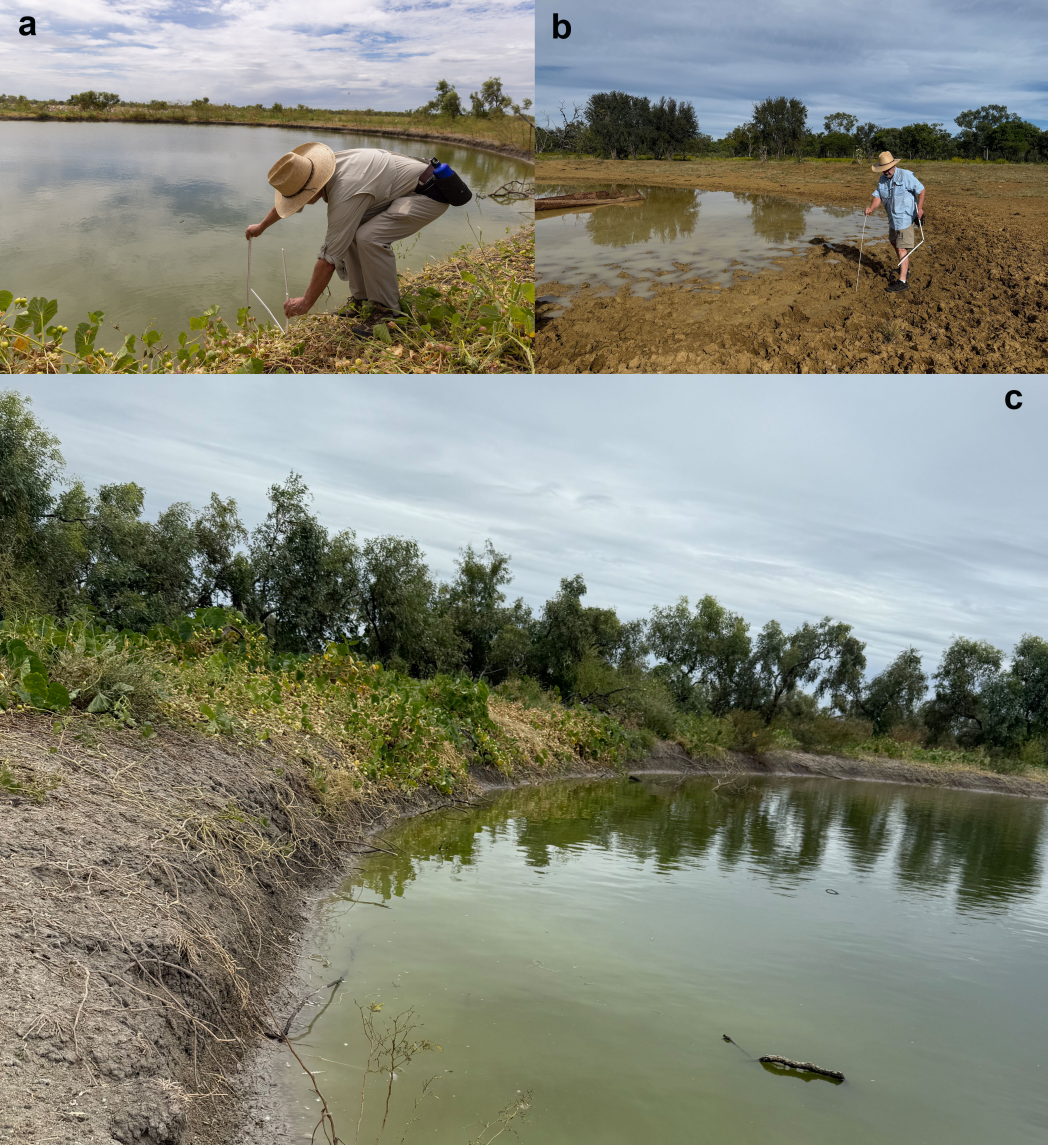
(a) Measuring slopes of banks surrounding a turkey-nest dam and (b) a nearby scrape; (c) a turkey-nest dam showing steep sides and lack of aquatic vegetation. Photographs by T.S.

The chief land use in this flat, dry area is raising of beef cattle [36]. The stock are watered in troughs connected via gravity-fed pipelines to raised (‘turkey-nest’) dams beside bores that are drilled to tap into artesian water (figure 2). These turkey-nest dams (TNDs) are separated by around 5 km on average and are broadly circular in shape with a diameter of approximately 30 m and a depth of 1−3 m (figure 2). The sloping sides of a TND are formed by bulldozing soil from a nearby site, resulting in many TNDs being adjacent to one or two shallow ‘scrapes’ that contain water for only part of the year. Water levels in TNDs are maintained by weekly checks and adjustments to bore flow (J. Dyer 2025, personal communication).

In this region, cane toads are found primarily around TNDs but also around anthropogenically watered areas (grounds of roadside hotels, farm buildings, etc.). We worked at three such artificially watered sites (HiWay Inn, Dunmarra Roadhouse, Newcastle Waters station homestead) and 25 TNDs (on Kalala, Hayfield-Shenandoah, Sturt Plains and Newcastle Waters stations). We gathered data over a 4-week period in September–October 2024 (late in the dry season) and a 3-week period in April–May 2025 (early in the dry season). Rainfall totals at Daly Waters during the survey period months were: September 2024 (28.0 mm); October 2024 (21.0 mm); April 2025 (19.4 mm); and May 2025 (9.0 mm) (https://www.eldersweather.com.au/).

### 2.3. Habitat attributes

Because the human development sites lacked open water, we scored habitat attributes only for TNDs and adjacent scrapes. To quantify slope of the land above and below the water’s edge at a TND or scrape, we recorded the height above water level at points 50 cm and 100 cm away from the shoreline (along an axis at 90° to the direction of the shoreline) and at the same distances and along the same axis in the water. We used folding metre-rules to establish the distances at which measurements were taken. The heights on land were taken by holding a metre-rule vertical at the water’s edge, and noting the height on that ruler intersected by a horizontally held ruler that rested on the bank either 50 cm or 100 cm away (figure 2). Likewise, we held a metre-rule vertically at 50 cm or 100 cm into the water from the shoreline, and recorded water depths at those points. These data were collected during the second (April–May 2025) trip.

### 2.4. Effects of amplexus on females

**Figure 3.**
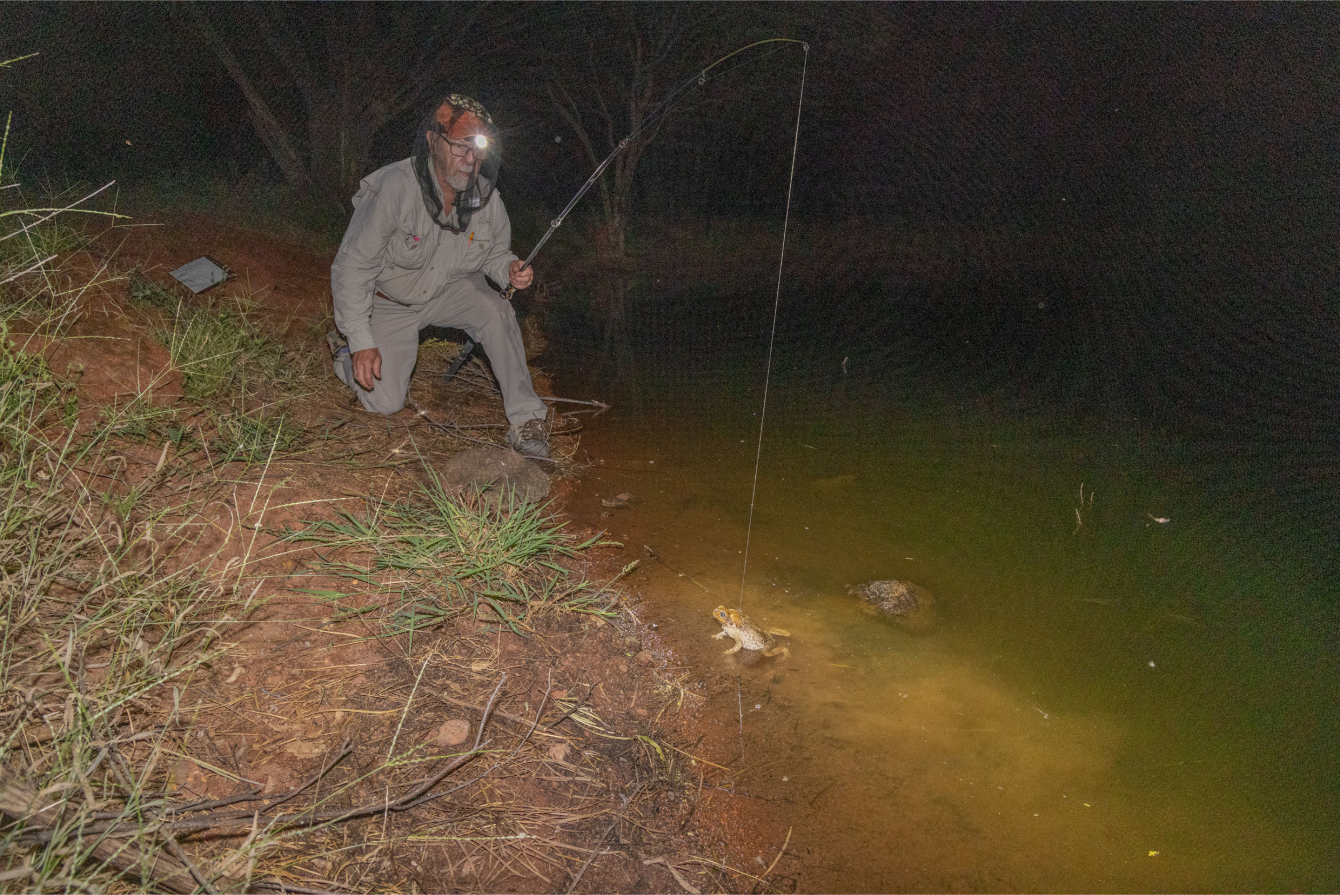
Method of inducing amplexus in free-ranging male cane toads by offering a tethered female. Photo by T.S.

We hand-captured adult female toads near TND 6 at night, fitted them with a cable-tie harness beneath the armpits and attached the harness to fishing line on a 3.5 m fishing rod (figure 3). Using red light to avoid disturbing the animals, we discreetly approached male toads at the water’s edge and dangled the female toad close to them. If the male approached and amplexed the female, we recorded (i) water depth and (ii) the proportion of time, over the next 30 seconds, that the female’s head was held underwater and hence, she was unable to breathe. We chose this short time period to avoid undue stress to the female. After that time, we lifted the pair above water to ensure that the female could breathe. Four adult females were used in the course of these trials. These data were collected on the second (April–May 2025) trip.

### 2.5. Orientations of male versus female toads

At a subsample of 12 TNDs and water-filled scrapes, we walked around the perimeter of the waterbody to score the sex and orientation of adult toads. Almost all toads were in the water, so we scored the direction in which the toad was facing relative to the shoreline. A toad in the water facing directly towards the shoreline was scored as 0°; an almost-direct alignment towards the shore would score as 10° or 350°; a toad facing directly away from the shoreline would score 180°. For analysis, we scored the absolute deviation of that angle from 0° (i.e. the resultant measure ranged from 0° to 180°). These data were collected on the second (April–May 2025) trip.

### 2.6. Abundance and operational sex ratios

On both trips, we visited each site at night (when adult toads are active [14,15,25]) and hand-collected toads for 30 minutes, both in the water and in surrounding terrestrial habitats (<50 m from the TND edge). We then measured each toad’s SUL (in mm) and body mass (g). Individuals > 80 mm SUL were classed as adult, and we determined the sex of those animals based on skin rugosity, colour, nuptial spines and the ‘release call’ [37]. We calculated OSR as the number of adult males per adult female [38]. We were unable to access four TNDs on the second trip because some roads were impassable.

### 2.7. Distribution of sexes around a pond

On the same site surveys as for §2.6 above, we recorded the habitat in which each adult toad was first encountered at that site, scored as ‘dam water’, ‘scrape water’ and ‘on land’. These data were collected on both field trips. We also scored distances along the perimeter of the dam between adjacent adult male toads.

### 2.8. Statistical analyses

Data were analysed using JMP Pro 18 and R version 4.3.1 [39]. Data were checked for normality and variance heterogeneity before analysis. We used linear regression to compare the amount of time that an amplexed female’s head was underwater (during the 30-second observation period) to water depth. To test whether orientation towards water differed by sex and waterbody type, we calculated the cosine of orientation angle (where 1 = directly facing the water, 0 = perpendicular, and −1 = directly facing away) and used this as the dependent variable. We first fitted a linear mixed-effects model with sex (male versus female), waterbody type (TND versus scrape) and their interaction as fixed effects. In this and other cases where we gathered data at multiple waterbodies, we included waterbody ID as a random factor to account for potential pseudoreplication. The interaction term was not statistically significant and was removed from the final model to improve interpretability and model parsimony. The final model included sex (male versus female) and waterbody type (TND versus scrape) as additive fixed effects and waterbody ID as a random effect. All models were fitted using the ‘lmer’ function from the *lme4* package in R [40], and *p*-values for fixed effects were obtained using Satterthwaite’s approximation via the *lmerTest* package [41]. We ln(1+X)-transformed data on average distance between adjacent adult males to achieve normality of distributions; and used this as the dependent variable in a one-factor ANOVA with pond type (TND versus scrape) as the independent predictor. To compare OSRs among habitats and survey seasons, we used OSR (% male) as the dependent variable in a two-factor ANOVA with habitat type (TND/scrape versus buildings) and year, and their interaction, as independent predictors. To compare habitat use between males and females, we used logistic regression with toad sex as the independent variable, and either survey season (late dry 2024 versus early dry 2025) or habitat type (land versus TND water versus scrape water) as the dependent variable (and including site as a factor).

All data used for this study are provided in the electronic supplementary material.

## 3. Results

### 3.1. Habitat attributes

**Figure 4.**
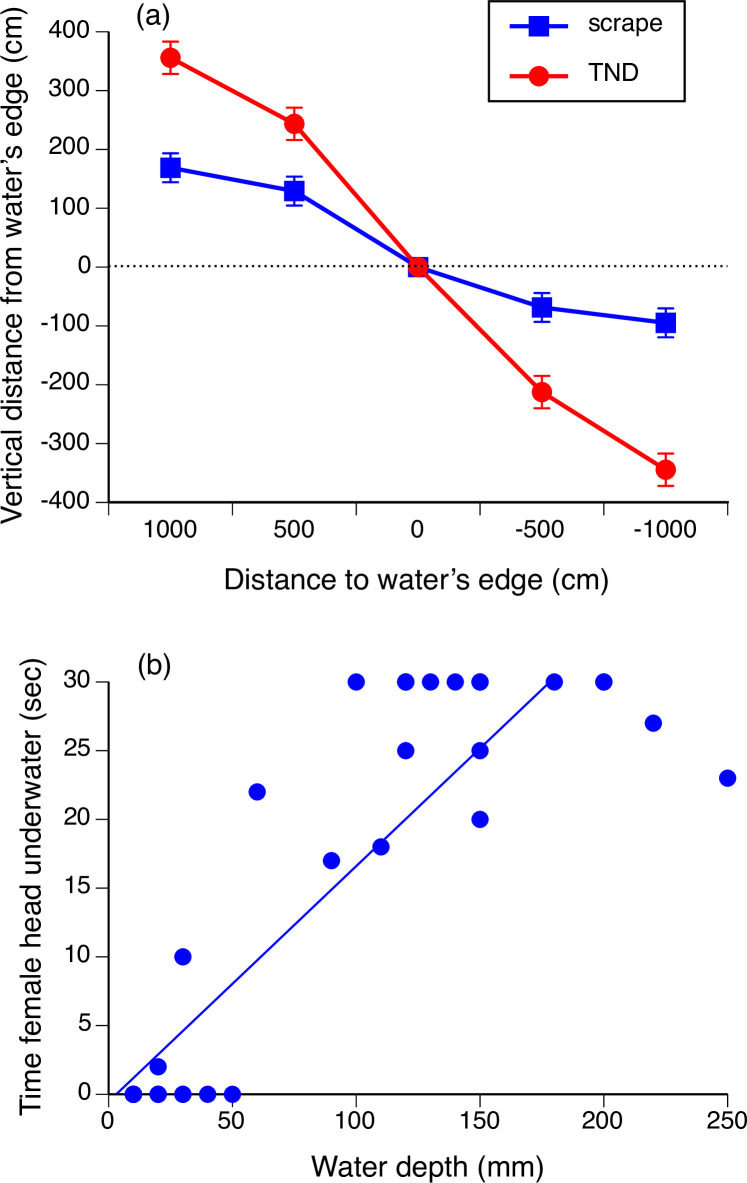
(a) Slopes of the banks above and below water for shallow scrapes and for turkey-nest dams (TNDs). The graph shows mean values and associated standard errors. (b) The amount of time (in a 30-second observation period) that a female cane toad’s head was held underwater by an amplexing male, as a function of water depth. Each data point shows a single trial.

The scrapes formed by removal of soil for dam-building were long, shallow depressions whereas TNDs had steep sides both above and below water level (figure 4a). The challenge to a toad of climbing back up the bank, and of finding shallow rather than deep water, was thus higher in TNDs than in scrapes.

### 3.2. Effects of amplexus on females

Under red light, free-ranging male toads readily approached and amplexed the tethered female (figure 3). The male’s attempt to seize the female often resulted in a vigorous struggle, with the female’s head held underwater. In our timed observations, females amplexed in water > 100 mm deep often were held underwater for the entire 30-second observation period whereas they kept their heads above water at shallower sites (linear regression *r*^2^ = 0.81, *t* = 13.95, 45 d.f., *p* < 0.0001; figure 4b).

Consistent with amplexus posing a drowning risk for females, we found one dead adult female (with no overt injuries) being amplexed by a male toad in a scrape (figure 1c) and found another dead (apparently drowned) female in a TND in a section of the dam where we had found freshly laid eggs 2 days previously. This was the only clutch of eggs and the only dead toad found in that TND during our surveys, consistent with drowning during amplexus.

### 3.3. Orientations of male versus female toads

**Figure 5.**
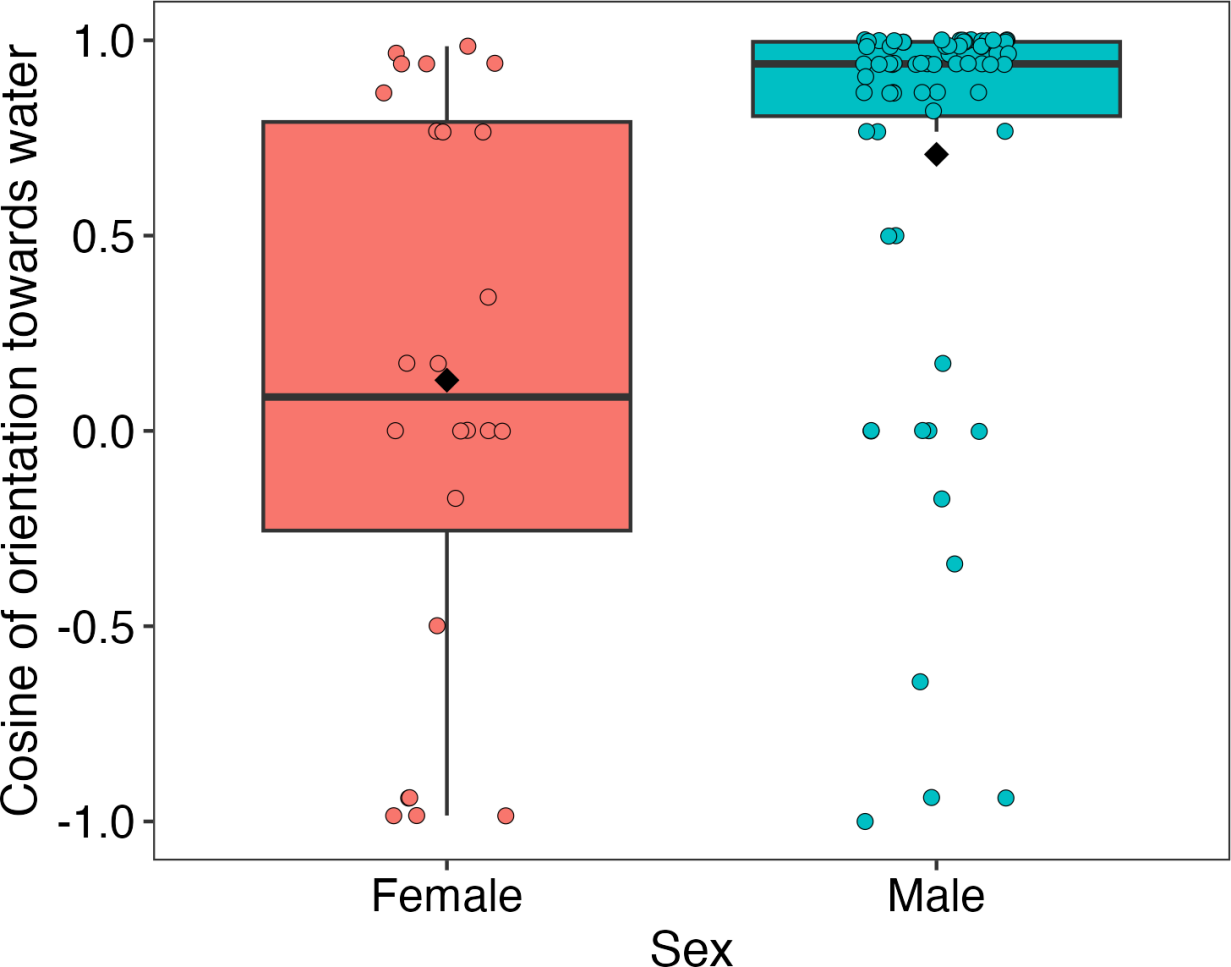
Orientation of free-ranging male and female cane toads relative to the edge of the waterbody in which they were found, based on 24 adult females and 68 adult males. Cosine-transformed orientation angles (where 1 = directly facing the water, 0 = perpendicular, and −1 = directly facing away) are shown by sex. Boxplots indicate the median and interquartile range; individual points represent raw data, with black diamonds denoting group means.

The orientation of toads towards the shoreline of waterbodies (cosine-transformed) differed significantly between sexes, with males more likely to face towards the water’s edge than were females (Estimate ± SE = 0.588 ± 0.146, *t* = 4.03, *p* < 0.001; figure 5). There was no significant difference in orientation based on waterbody type (TND versus scrape; Estimate ± SE = 0.111 ± 0.174, *t* = 0.64, *p* = 0.552), and no evidence of an interaction between sex and waterbody type (interaction term *p* = 0.24; not included in final model).

### 3.4. Abundance and operational sex ratios

Total numbers of adult toads collected per site per trip differed among surveys and sites, with average values of 111.3 (SE = 35.6) and 92.5 (SE = 12.3) for buildings and TNDs/scrapes in 2024, respectively; and corresponding figures of 28.0 (35.6) and 79.0 (13.5) in 2025. The range overall was from 6 to 305 toads. At the 12 sites for which we scored toad orientations in 2025 (see above), the average distance between adjacent adult males along the shoreline was 1.59 m (SE = 0.16) at TNDs and 3.63 m (SE = 1.08) at scrapes (not significantly different; ANOVA on ln(1+X)-transformed data, with pond type as factor, *F*_1,67_ = 0.13, *p* = 0.78).

The OSR was highly male-biased around TNDs/scrapes (mean = 75% male in the late dry season of 2024, 65% male in the early dry season of 2025) whereas that around buildings was female-biased (44% male in the late dry season of 2024, 40% in the early dry season of 2025). The difference in OSR between TNDs/scrapes versus buildings was statistically significant (*F*_1,24.2_ = 15.81, *p* < 0.0006) whereas the difference between survey seasons was not (*F*_1,22.5_ = 2.42, *p* = 0.13; interaction *F*_1,22.5_ = 0.40, *p* = 0.53).

### 3.5. Distribution of sexes around a pond

**Figure 6.**
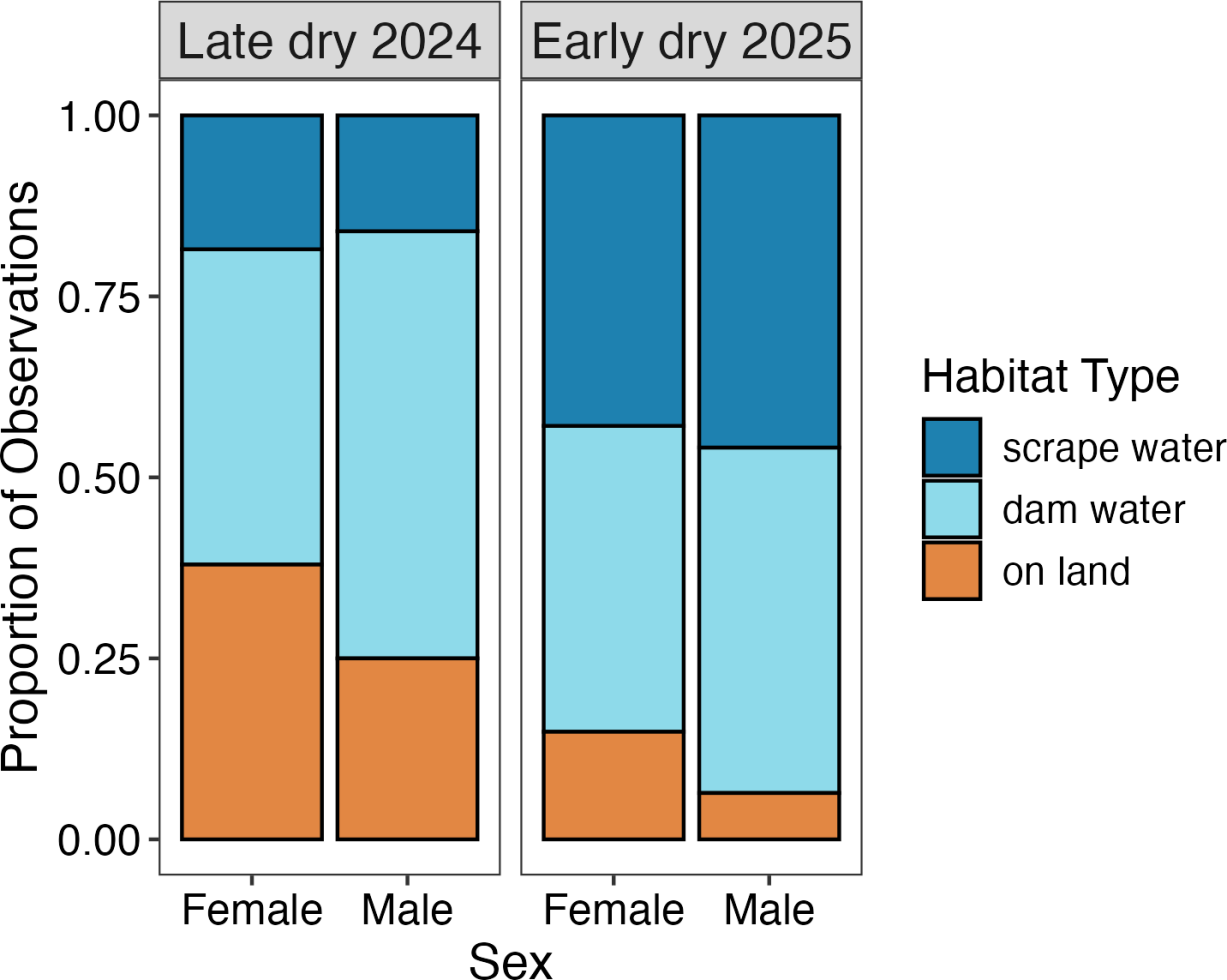
Proportional habitat use by male and female cane toads during the late dry season 2024 and early dry season 2025. Stacked bars show the proportion of observations for each sex in different habitat types: on land, in dam water and in scrape water. Data are separated by season to illustrate seasonal variation in habitat use.

The relative numbers of toads captured in each of the three habitat types (on land, in dam water and in scrape water) differed between the two field trips. During the late dry season in September–October 2024, 37% of a total of 3881 toads were found on land, compared to 13% of 2017 toads in the early dry season of April–May 2025. Corresponding proportions for dam water were 55 and 46% and for scrape water 17 and 45%. That is, toads were more likely to be in scrape water, and less likely to be on land, during the second trip (early dry season 2025; logistic regression χ^2^ = 1099.61, 2 d.f., *p* < 0.0001). On both trips, the use of these three habitat types differed between males and females. Restricting the analysis to sites surveyed at times when scrapes contained water (and thus were available for toad rehydration; *n* = 2122 toads), females were found on land more often than were males (38% versus 25% in late dry 2024; 15% versus 6% in early dry 2025), less often in dam water (44% versus 59% in late dry 2024; 42% versus 48% in early dry 2025), and more often in scrape water in the late dry (18% versus 16% in late dry 2024), but less often in scrape water than males in the early dry (43% versus 46% in early dry 2025; figure 6). A logistic regression with toad sex as the independent variable and habitat use as the dependent variable (and including site as a factor) showed that males and females differed in habitat use during both surveys (late dry 2024: χ^2^ = 29.40, 2 d.f., *p* < 0.0001; early dry 2025, χ^2^ = 19.93, 2 d.f., *p* < 0.0001). Broadly, most adult males were found in dam water, particularly in the late dry, and most female toads were found either on land or, early in the dry season, in shallow scrape water. Overall, females were nearly twice as likely to be found on land than males (figure 6).

## 4. Discussion

Sexual segregation in habitat use is widespread in anuran amphibians, as in many types of animals [1], but the spatial separation between males and females is amplified by the scarcity of hydration opportunities at the arid-zone fringe of the cane toad’s invasion of Australia. For most of the year, the only places that a cane toad can survive in our study area are either around farm dams (TNDs and scrapes) or several kilometres away around artificially irrigated areas associated with remote anthropogenic infrastructure (e.g. roadhouses and homesteads, with typically >50 km between such infrastructure). Broadly, more male toads were found around dams whereas more females were found around buildings. Even in the area close to a dam, females used open water less often than did males. The degree of spatial separation between the sexes thus is higher in these semi-arid environments than in mesic habitats within the species’ Australian range [12,13].

Our data are consistent with the idea that female toads avoid areas with high male densities because of the costs of harassment. For a male cane toad, the risk of prolonged amplexus and drowning is minimal because males give a ‘release call’ that stimulates amplectant male toads to let go [42]. However, females cannot give the release call, and (although they often vibrate when seized) they have limited ability to terminate amplexus [43] (but see also [44]). Steep sides to TNDs render an amplexed female likely to find herself in deep water, and unable to keep her head above water or to clamber to safety, as evidenced by our amplexus trials in TNDs and discovery of putatively drowned females. The scarcity of alternative hydration sites imposes a stark choice for an adult female cane toad: she can survive the dry season in relative safety around anthropogenically disturbed sites, but these provide no open waterbodies, and thus no opportunity to spawn; or she can shelter near a dam that provides that breeding access but at the cost of harassment whenever she needs to rehydrate.

Similar trade-offs likely are common in areas with seasonal regimes of temperature and precipitation: for example, male Arafura filesnakes (*Acrochordus arafurae*) must choose whether to spend the dry season in deep-water billabongs that provide opportunities to mate versus shallow-water billabongs that provide opportunities to feed [45]. As in the case of the cane toads in the current study, the individuals must make that choice during the wet season, when movement between alternatives is possible. Radio-tracking at our study sites even during relatively favourable hydric conditions (early dry season) revealed high mortality rates of cane toads attempting to cross the relatively short distances (500 m) between adjacent moist sites (C.J.J., unpublished data). Because of high rates of cannibalism and pheromonal suppression of eggs and hatchlings by older toad tadpoles [46], there may be a massive fitness benefit to being the first to breed in any given waterbody—an option likely unavailable to a female toad that spends the dry season kilometres away in an artificially irrigated area. Alternatively, a female toad might be able to survive the long dry season by finding moist shelter in a natural drainage channel, closer to a dam; but in an unusually dry year such a site may become lethally arid before the reprieve of the next wet season. More generally, the cost to escaping harassment may be increased vulnerability to other dangers. Juvenile female red-sided gartersnakes (*Thamnophis sirtalis parietalis*) avoid harassment by males at communal overwintering dens by dispersing in cold weather, when males are inactive—but at the cost of high mortality from predatory crows [47].

The danger to female toads at TNDs may be increased by intense sexual selection on males. Given few opportunities to breed each year (because larval cannibalism reduces the number of successful clutches per pond per year [46]), males in semi-arid habitats may be under intense selection to amplex any potential mate. By restricting successful reproduction to a small proportion of males each year, selection around TNDs may favour less discriminating and more prolonged amplexus than would be the case in a mesic area with abundant ephemeral wet-season ponds. The behaviour and spatial distribution of males at TNDs may intensify this selection. Males were regularly spaced around the edges of both scrapes and dams, facing the bank and ready to intercept any incoming females, including ones offered to them during our experiments. Hence, arid conditions may intensify sexual conflict by changing male behaviour as well as by restricting spawning opportunities for females to sites that entail high risks of harassment. Long-term mark–recapture studies could usefully evaluate the possibility that conflict-driven mortality creates a feedback loop by incrementally reducing the numbers of females relative to males, further intensifying that conflict (see [48] for an example with lizards).

Because cane toads invaded our study area recently (about 15 years ago; J. Dyer 2025, personal communication), novel evolutionary pressures have had only a few generations to modify male and female behaviours. Consequently, the spatial separation between males and females likely reflects phenotypically plastic effects on pre-existing sex-based behavioural divergences, rather than recently evolved adaptations. Our study adds to the range of novel challenges that invasive toads have encountered in the course of their range expansion into conditions that are drier and hotter than in their native range [27]. Extensive studies have documented evolutionary responses to aridity and higher temperatures, as well as to increased abundance of conspecifics and thus intraspecific competition [46,49] as well as to pressures for enhanced rates of dispersal [50]. Likewise, the invasion into arid areas has restricted opportunities for breeding to TNDs: sites with novel physical aspects (bank steepness), biological attributes (lack of floating vegetation) and spatial connectedness (low proximity to other mesic areas). One result of that situation has been to skew OSRs strongly—further exacerbating the potential for sexual harassment to imperil female toads. In this sense, the toad’s expansion into arid habitats has influenced patterns of metapopulation demography as well as mating systems.

Mathematical models for species without paternal care (as in cane toads) predict that the rate of population growth depends more on the number of females than of males because a single male can fertilize the eggs of multiple females [51]. Ideally, then, attempts to cull invasive species should target times and places where females are most abundant (in this case, around buildings rather than around TNDs). If high OSRs around dams increase mortality rates of females (as seems plausible from our study, and has been reported in experimental studies on lizards [48]), culling male toads around dams might increase rather than decrease total reproductive output from that system. That is, toad control might usefully focus on manipulating OSRs at breeding sites rather than attempting to remove all toads. Experimental manipulation of OSRs around dams, to directly evaluate the impacts on mortality rates of females, would be of great interest. Effects may be trivial, if most females can achieve hydration without risk of drowning; but a highly skewed OSR may increase the incidence of multiple-male amplexus of females, further increasing the risk of drowning. Exploiting intraspecific competitive mechanisms for pest control has already worked well for cane toads, via attracting cannibalistic larvae to pheromone-baited funnel-traps [52] or using such larvae to eliminate newly laid eggs (R.S., in preparation). Intense sexual conflict within arid-zone toad populations may render the same approach useful with reproductive behaviour. Removing almost all males is easy to do but will have no effect on recruitment, so long as even a few males persist; but selectively culling females might reduce survivorship of the few surviving female toads by exacerbating sexual conflict.

## Data Availability

All data are available in the electronic supplementary material [53].
